# Supplementary material for: Evaluating the effect of Bolsa Familia, Brazil’s conditional cash transfer programme, on maternal and child health: A study protocol
Source: PLoS One. 2022 May 23;17(5):e0268500. doi: 10.1371/journal.pone.0268500 (PMC9126365; doi:10.1371/journal.pone.0268500)
Supplement: S1 File — (DOCX) [file pone.0268500.s001.docx]

**Chart 1.** Characteristics for specific objectives.

| **Secondary objective** | **Data source** | **Period** | **Exposure to BFP** | **Variables for propensity score (PS)** |
| --- | --- | --- | --- | --- |
| To evaluate the effect of BFP on birth weight | Data from the “100 Million Brazilian Cohort” linked to SINASC | 2004 to 2015 | The exposure is defined as having started receiving BFP before the birth of their child in the 2004 to 2015 period and did not stop receiving from pregnancy to delivery. | 1. Sociodemographic characteristics:  Mother education (8 years or more of study /4 to 7 years / ≤3 years), race/ethnicity (white / black / Asian descend / mixed / indigenous), marital status (with partner / with no partner).  2. Household characteristics:  Location of household (urban/rural), household building material (brick or rubble/other), water supply (public network/other), electricity (meter of private or community use/no meter), garbage collection (yes/no), sanitary sewage (public network/other), overcrowded (yes/no), year of registry. |
| To evaluate the effect of BFP on small and large for gestational age (SGA/LGA) | Data from the “100 Million Brazilian Cohort” linked to SINASC | 2012 to 2015 | The exposure is defined as having started receiving BFP before the birth of their child in the 2012 to 2015 period and did not stop receiving from pregnancy to delivery. | 1. Sociodemographic characteristics:  Mother education (8 years or more of study /4 to 7 years / ≤3 years), race/ethnicity (white / black / Asian descend / mixed / indigenous), marital status (with partner / with no partner).  2. Household characteristics:  Location of household (urban/rural), household building material (brick or rubble/other), water supply (public network/other), electricity (meter of private or community use/no meter), garbage collection (yes/no), sanitary sewage (public network/other), overcrowded (yes/no), year of registry. |
| To evaluate the effect of BFP on preterm birth (PTB) | Data from the “100 Million Brazilian Cohort” linked to SINASC | 2012 to 2015 | The exposure is defined as all live births whose mothers received the BFP continuously throughout the pregnancy period, i.e., from the gestation start date (calculated by the difference between child's birth date and gestational age) to the birth date. | 1. Sociodemographic characteristics:  Cohort time (≥5 / <5 years, calculated by the difference between child's birth date and data of mothers entering the Cohort), self-reported maternal race/skin color (white/black+mixed/indigenous).  2. Household characteristics:  Geographical region (South/North/Northeast/Southeast/ Midwest), location of household (urban/rural), household building material (brick or rubble*/*other), sewage (public network/other), water supply (public network/other), garbage disposal (city collection/no collection-burned or buried), electricity (meter of private or community use/no meter), and household density (≤2 */* >2 people per room, calculated by dividing the number of individuals living in the house and the number of rooms). |
| To evaluate the effect of BFP on maternal mortality | Data from the “100 Million Brazilian Cohort” linked to SINASC and SIM | 2004 to 2015 | The exposure is defined as having started receiving BFP before or during the birth of their child in the 2004 to 2015 period and did not stop receiving before the outcome or until childbirth. | 1. Sociodemographic characteristics:  Mother education (8 years or more of study / 4 to 7 years / ≤3 years), race/ethnicity (white / black / Asian descend / mixed / indigenous), maternal age, number of childbirth registered in the cohort.  2. Household characteristics:  Location of household (urban/rural), household building material (brick or rubble/other), water supply (public network/other), electricity (meter of private or community use/no meter), garbage disposal (city collection/no collection-burned or buried), sanitary sewage (public network/other), overcrowded (yes/no), year of registry. |
| To evaluate the effect of BFP on child malnutrition | Data from the “100 Million Brazilian Cohort” linked to SISVAN and SINASC | 2008 to 2017 | Children who started and did not stop receiving the BFP before the last visit. | 1. Sociodemographic characteristics:  Mother’s characteristics: Mother education (8 years or more of study / 4 to 7 years / ≤3 years), race/ethnicity (white /black /Asian descend / mixed / indigenous), marital status (with partner / with no partner).  2. Household characteristics:  Location of household (urban/rural), household building material (brick or rubble/other), water supply (public network/other), electricity (meter of private or community use/no meter), garbage disposal (city collection/no collection-burned or buried), sanitary sewage (public network/other), overcrowded (yes/no), year of registry. |
