# Supplementary material for: Evaluating the effect of Bolsa Familia, Brazil’s conditional cash transfer programme, on maternal and child health: A study protocol
Source: PLoS One. 2022 May 23;17(5):e0268500. doi: 10.1371/journal.pone.0268500 (PMC9126365; doi:10.1371/journal.pone.0268500)
Supplement: Attachment — Submitted filename: Asw_2rev_19042022.docx [file pone.0268500.s003.docx]

To the Editor and reviewers,

First, we would like to express our appreciation for your consideration of our manuscript. We are very grateful for the pertinent criticism offered, and believe the incorporation of the reviewer’s suggestions will greatly contribute to the quality of our publication. Please find our point-by-point responses below to the criticism raised by each reviewer:

Journal Requirements:

Please review your reference list to ensure that it is complete and correct. If you have cited papers that have been retracted, please include the rationale for doing so in the manuscript text, or remove these references and replace them with relevant current references. Any changes to the reference list should be mentioned in the rebuttal letter that accompanies your revised manuscript. If you need to cite a retracted article, indicate the article’s retracted status in the References list and also include a citation and full reference for the retraction notice.

- We appreciate the suggestion. We have carefully reviewed our list of references. We did not have articles with retractions. We included newer government decrees, but kept those in force in the period defined by the study.

[Note: HTML markup is below. Please do not edit.]

Reviewers' comments:

Reviewer's Responses to Questions

**Comments to the Author**

1. Does the manuscript provide a valid rationale for the proposed study, with clearly identified and justified research questions?

The research question outlined is expected to address a valid academic problem or topic and contribute to the base of knowledge in the field.

Reviewer #1: Yes

Reviewer #2: Yes

Reviewer #3: Yes

2. Is the protocol technically sound and planned in a manner that will lead to a meaningful outcome and allow testing the stated hypotheses?

The manuscript should describe the methods in sufficient detail to prevent undisclosed flexibility in the experimental procedure or analysis pipeline, including sufficient outcome-neutral conditions (e.g. necessary controls, absence of floor or ceiling effects) to test the proposed hypotheses and a statistical power analysis where applicable. As there may be aspects of the methodology and analysis which can only be refined once the work is undertaken, authors should outline potential assumptions and explicitly describe what aspects of the proposed analyses, if any, are exploratory.

Reviewer #1: Yes

Reviewer #2: Yes

Reviewer #3: Yes

3. Is the methodology feasible and described in sufficient detail to allow the work to be replicable?


Descriptions of methods and materials in the protocol should be reported in sufficient detail for another researcher to reproduce all experiments and analyses. The protocol should describe the appropriate controls, sample size calculations, and replication needed to ensure that the data are robust and reproducible.

Reviewer #1: Yes

Reviewer #2: Yes

Reviewer #3: Yes

4. Have the authors described where all data underlying the findings will be made available when the study is complete?

The [PLOS Data policy](https://journals.plos.org/plosone/s/materials-and-software-sharing) requires authors to make all data underlying the findings described in their manuscript fully available without restriction, with rare exception, at the time of publication. The data should be provided as part of the manuscript or its supporting information, or deposited to a public repository. For example, in addition to summary statistics, the data points behind means, medians and variance measures should be available. If there are restrictions on publicly sharing data—e.g. participant privacy or use of data from a third party—those must be specified.

Reviewer #1: Yes

Reviewer #2: Yes

Reviewer #3: Yes

5. Is the manuscript presented in an intelligible fashion and written in standard English?

PLOS ONE does not copyedit accepted manuscripts, so the language in submitted articles must be clear, correct, and unambiguous. Any typographical or grammatical errors should be corrected at revision, so please note any specific errors here.

Reviewer #1: Yes

Reviewer #2: Yes

Reviewer #3: Yes

6. Review Comments to the Author

Please use the space provided to explain your answers to the questions above and, if applicable, provide comments about issues authors must address before this protocol can be accepted for publication. You may also include additional comments for the author, including concerns about research or publication ethics.

You may also provide optional suggestions and comments to authors that they might find helpful in planning their study.


(Please upload your review as an attachment if it exceeds 20,000 characters)

Reviewer #1: Overall, the manuscript is much more clear. The authors have made important improvements. There are, however, a few remaining issues to be addressed:

1- Please check your acronyms. Sometimes you use PBF, and other times BFP.

- We appreciate your careful review. We revised the text and changed the acronym that was present in reference 15, line 449.

2- Line 158-159: Why drop babies of less than 500g or born before 22 gestational weeks? I know they may be less likely to survive but could extreme prematurity and/or very low birthweight not be related to the socioeconomic standing of the mother? Is this not conditioning on the outcome? I understand the rationale for multiple births or congenital abnormalities as they are unlikely to be related to SES but related to birthweight and prematurity, but excluding the babies based on their likelihood of survival does not make sense to me.

- We appreciate your comment. We consider that survival may be related to SES and better access to a specialized delivery service, we used this feasibility criterion (<500g and <22sg). As pointed out, women with better SES could be more likely to have babies with >300g or >20sg (criteria used in developed countries), but we must emphasize that we are working with populations of socioeconomically vulnerable women. In a way, we agree with your comment and removed the mention of the criterion that will be applied, keeping only the phrase “Fetal viability criteria can be applied [62-65]. "

3- On line 241, do you mean the parent's education or the child's?

- We appreciate the comment. We apologize, but we could not find this passage in the text. When we refer to education in the text, we are usually referring to maternal schooling. Except in some passages of the text (when citing the BFP conditionalities), we refer to school attendance by adolescents (<17).

4- Line 270: You say "quasi-experimental approaches". You should explain how your approaches are quasi-experimental (i.e. exogenous changes with the BF program and subsequent outcomes, or regression discontinuity designs around the cut points for BF eligibility). I'm not sure I would use this characterization here based on how your have described your proposed analyses.

- We appreciate your comment and agree that the term used was not so appropriate. We rewrite the sentence to “This study will use propensity score-based methods to assess the BFP effect on maternal and child health outcomes in a large sample of poor and impoverished Brazilian households.”

Reviewer #2: The authors addressed all the points raised. I believe that this work have the potential to improve methodological rigor in future researches exploring maternal and child health outcomes

- We appreciate your careful review.

Reviewer #3: Thank you for the clarifications and revisions. In finalizing, I suggest you consider reconsidering words like "effect" and "impact" if you wish to be more careful around causal language (see you response to my first comment). In chart 1, column 1 it often says "access" but I believe you mean "assess"

- We appreciate your careful review. We consider maintaining the term “effect”. We consider this to be justified by the limitations of each study generated from this protocol. We know that we cannot sustain the “causal effect” and that the effect of the Bolsa Família found will probably be associative, due to the characteristics of the program and the proposed analysis (adequate to the particularities of the program and the database).

- We have modified the terms in Chart 1 to “To evaluate”.

7. PLOS authors have the option to publish the peer review history of their article ([what does this mean?](https://journals.plos.org/plosone/s/editorial-and-peer-review-process#loc-peer-review-history)). If published, this will include your full peer review and any attached files.


If you choose “no”, your identity will remain anonymous but your review may still be made public.


**Do you want your identity to be public for this peer review?** For information about this choice, including consent withdrawal, please see our [Privacy Policy](https://www.plos.org/privacy-policy).

Reviewer #1: No

Reviewer #2: No

Reviewer #3: **Yes:** John A. Maluccio

[NOTE: If reviewer comments were submitted as an attachment file, they will be attached to this email and accessible via the submission site. Please log into your account, locate the manuscript record, and check for the action link "View Attachments". If this link does not appear, there are no attachment files.]

While revising your submission, please upload your figure files to the Preflight Analysis and Conversion Engine (PACE) digital diagnostic tool, <https://pacev2.apexcovantage.com/>. PACE helps ensure that figures meet PLOS requirements. To use PACE, you must first register as a user. Registration is free. Then, login and navigate to the UPLOAD tab, where you will find detailed instructions on how to use the tool. If you encounter any issues or have any questions when using PACE, please email PLOS at [figures@plos.org](mailto:figures@plos.org). Please note that Supporting Information files do not need this step.

- We appreciate the suggestion. The figure was generated from the PACE digital diagnostic tool and attached to this submission.
